# Effects of intima stiffness and plaque morphology on peak cap stress

**DOI:** 10.1186/1475-925X-10-25

**Published:** 2011-04-08

**Authors:** Ali C Akyildiz, Lambert Speelman, Harald van Brummelen, Miguel A Gutiérrez, Renu Virmani, Aad van der Lugt, Anton FW van der Steen, Jolanda J Wentzel, Frank JH Gijsen

**Affiliations:** 1 Department of Biomedical Engineering, Thoraxcenter, Erasmus Medical Center, Rotterdam, the Netherlands; 2 Interuniversity Cardiology Institute of the Netherlands (ICIN), Utrecht, the Netherlands; 3 Department of Mechanical Engineering, Eindhoven University of Technology, Eindhoven, the Netherlands; 4 Department of Mathematics and Computer Science, Eindhoven University of Technology, Eindhoven, the Netherlands; 5 Faculty of Mechanical, Maritime and Materials Engineering, Delft University of Technology, Delft, the Netherlands; 6 CVPath Institute Inc., Gaithersburg, MD, USA; 7 Department of Radiology, Erasmus Medical Center, Rotterdam, the Netherlands

## Abstract

**Background:**

Rupture of the cap of a vulnerable plaque present in a coronary vessel may cause myocardial infarction and death. Cap rupture occurs when the peak cap stress exceeds the cap strength. The mechanical stress within a cap depends on the plaque morphology and the material characteristics of the plaque components. A parametric study was conducted to assess the effect of intima stiffness and plaque morphology on peak cap stress.

**Methods:**

Models with idealized geometries based on histology images of human coronary arteries were generated by varying geometric plaque features. The constructed multi-layer models contained adventitia, media, intima, and necrotic core sections. For adventitia and media layers, anisotropic hyperelastic material models were used. For necrotic core and intima sections, isotropic hyperelastic material models were employed. Three different intima stiffness values were used to cover the wide range reported in literature. According to the intima stiffness, the models were classified as stiff, intermediate and soft intima models. Finite element method was used to compute peak cap stress.

**Results:**

The intima stiffness was an essential determinant of cap stresses. The computed peak cap stresses for the soft intima models were much lower than for stiff and intermediate intima models. Intima stiffness also affected the influence of morphological parameters on cap stresses. For the stiff and intermediate intima models, the cap thickness and necrotic core thickness were the most important determinants of cap stresses. The peak cap stress increased three-fold when the cap thickness was reduced from 0.25 mm to 0.05 mm for both stiff and intermediate intima models. Doubling the thickness of the necrotic core elevated the peak cap stress by 60% for the stiff intima models and by 90% for the intermediate intima models. Two-fold increase in the intima thickness behind the necrotic core reduced the peak cap stress by approximately 25% for both intima models. For the soft intima models, cap thickness was less critical and changed the peak cap stress by 55%. However, the necrotic core thickness was more influential and changed the peak cap stress by 100%. The necrotic core angle emerged as a critical determinant of cap stresses where a larger angle lowered the cap stresses. Contrary to the stiff and intermediate intima models, a thicker intima behind the necrotic core increased the peak cap stress by approximately 25% for the soft intima models. Adventitia thickness and local media regression had limited effects for all three intima models.

**Conclusions:**

For the stiff and intermediate intima models, the cap thickness was the most important morphological risk factor. However for soft intima models, the necrotic core thickness and necrotic core angle had a bigger impact on the peak cap stress. We therefore need to enhance our knowledge of intima material properties if we want to derive critical morphological plaque features for risk evaluation.

## Background

**Figure 1:**
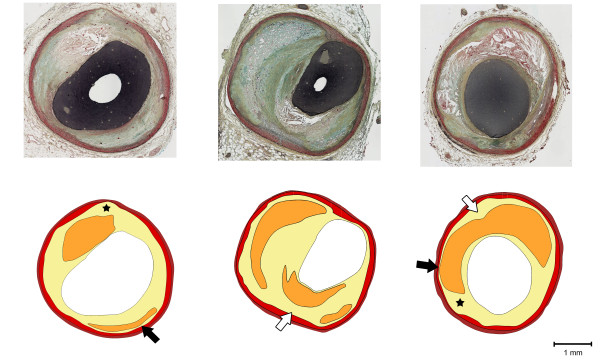
**Examples of coronary artery plaques having different geometric features: histological cross-sections (upper panel), and corresponding color-coded, manually drawn contours (lower panel)**. Key to the colors used: brown = adventitia, red = media, yellow = intima, and orange = necrotic core. White arrows indicate the locations with a thick intima layer and black arrows indicate the locations with a thin intima layer behind the necrotic core. Stars show severely compromised media.

Atherosclerosis is a cardiovascular disease that is characterized by local thickening of the vessel wall, or plaque formation. A subset of atherosclerotic plaques, called vulnerable plaques, is characterized by lipid accumulation in the vessel wall, with a thin fibrous cap separating the necrotic core from the lumen (figure 1) [1]. Rupture of the cap of a vulnerable plaque in a coronary artery is the underlying cause of the majority of acute myocardial infarctions and sudden coronary deaths [2,3].

Rupture of a cap occurs when the mechanical stress in the cap exceeds its strength. The determinants of the stress distribution in a plaque are the loading conditions, the plaque geometry and the material properties of the plaque constituents. Variations in these factors affect the stress values and the stress distribution in the cap significantly. Therefore, detailed investigations of these features are essential to reveal biomechanical risk factors for plaque rupture.

The possible role of local mechanical stress as a predictor for plaque rupture [4-6] instigated many studies to explore the effects of various geometric plaque features on the cap stresses. Some studies concentrated on real plaque geometries obtained from intravascular ultrasound [7,8], magnetic resonance [9,10], or histology images [11-15] whereas others used idealized geometries [16-19]. The influence of the cap thickness has been investigated most extensively. In several computational studies, it was demonstrated that the stresses in the cap increase exponentially with decreasing cap thickness [8,9,18]. However, the large variation of cap thickness of ruptured plaques [20] indicates that cap thickness is not the only relevant geometric plaque feature. This was illustrated in a comprehensive parameter study by Ohayon et al. [16]. They showed that the size of the necrotic core also has a significant influence on cap stresses, and speculated that the thickness of the lipid core may be as critical for plaque rupture as the cap thickness.

It is likely that besides cap thickness and necrotic core thickness, other geometric features play a role in plaque rupture. Structures behind the necrotic core, including intima, media and adventitia tissue, might influence cap stresses as well. These plaque components have diverse morphological structures. The thickness of the intima tissue can vary greatly. Some necrotic cores are separated from the media by a thick layer of tissue, while others almost touch the media (figure 1). The thickness of the media also shows great variation: at some locations, the media is severely compromised or even completely degraded (figure 1).

The material properties of plaque components are determined by their composition, which might vary greatly among different plaques. The intima tissue is especially heterogeneous: it consists of extracellular proteoglycan-rich matrix material, smooth muscle cells, inflammatory cells, collagen etc. [20]. Since these components are present in different amounts in different plaques, the material properties of the intima tissue may vary greatly. Experimental studies on atherosclerotic plaque material properties have mostly reported high intima stiffness with the Young's moduli (*E*) between 500 kPa and 1000 kPa, even up to 2300 kPa [19,21-26]; *E *= 1000 kPa has frequently been used in the numerical studies [7,12,18]. However, Lee et al. measured much lower *E *values: an average of 41 kPa for the nonfibrous and 82 kPa fibrous atherosclerotic intima tissues [27]. This finding has been supported by a recent study that used an advanced testing method and reported a mean E value of 33 kPa[28].

The aim of our study was to investigate the influence of the variation of intima material properties and of geometric variations of different plaque components on cap stresses. A parametric study was carried out to explore the individual and combined effects of possible biomechanical determinants of plaque rupture.

## Methods

Two dimensional (2D) idealized geometries of varying cap thickness, necrotic core thickness and angle, intima thickness behind the necrotic core, adventitia thickness, and local regression depth in the media tissue were generated to mimic the different geometric features of atherosclerotic plaque cross-sections. Different stiffness values for the intima were used in the models such that the stiffness range reported in literature [21,22,24-28] was covered to explore its influence on peak cap stress.

### Idealized geometries

**Figure 2:**
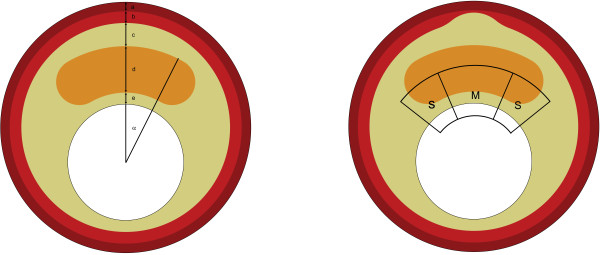
**Examples of computational models. **The model on the left panel shows different geometric features: α= Necrotic core angle, a= Adventitia thickness, b= Media thickness, c= Intima thickness behind the necrotic core, d= Necrotic core thickness, and e= Cap thickness. The model on the right panel shows local media regression in the center. S indicates shoulder region and M indicates midcap region. Key to the colors used: brown=adventitia, red=media, yellow= intima, and orange=necrotic core.

The symmetric idealized baseline geometry (figure 2.A) was constructed based on in vivo imaging studies [7,18,29-33] and morphometric analysis of histology images obtained from 10 atherosclerotic human coronaries. The structure comprises five different geometric components: cap, necrotic core, intima, media and adventitia. The cap was defined as the region of the intima separating the necrotic core and the lumen. By varying the geometric parameter values (Table 1), a large set of different 2D idealized plaque models was obtained. The lumen diameter was kept constant in all models. The thickness of each geometric component, except the media layer, was altered uniformly. The thickness of each geometric component, except the media layer, was altered uniformly. The media layer of an atherosclerotic plaque often shows local regression rather than uniform thinning. To model local media regression (figure 1, black arrows), the thickness of the media layer was reduced at the center or shoulder region (figure 2.B). The necrotic core angle was defined as given in figure 2.A.

**Table 1:** Baseline values and ranges of the geometric features used in computational models

Geometric feature	Baseline Value	Value Range (:Increment)
Lumen radius	1.25 mm	-
Cap thickness	0.05 mm	0.05 - 0.25 (:0.05) mm
Necrotic core thickness	1.20 mm	0.60 - 1.40 (:0.2) mm
Necrotic core angle	30°	10° - 40° (:10°)
Thickness of intima layer behind the necrotic core	0.50 mm	0.30 - 0.70 (:0.1) mm
Media thickness	0.25 mm	0.05 - 0.25 (:0.05) mm
Adventitia thickness	0.15 mm	0.10 - 0.20 (:0.05) mm

Thickness of all sections but the media was altered uniformly. For the media section, the values are the thickness values at the location of media regression.

### Material properties

Four different tissue types were used as plaque constituents: intima, necrotic core, media, and adventitia. Intima material properties were also applied to the cap. All tissue types were assumed to be incompressible and homogenous.

The neo-Hookean material model [34] was employed for the intima and necrotic core:(1)

where *W*_*NH *_[N/m^2^] *is *the strain energy density function and I_1 _= λ_r_^2^+ λ_θ_^2+ ^λ_z_^2 ^is the first invariant with λ_r_, λ_θ_, and λ_z _being the principal stretches in the radial, circumferential and axial directions. The only material constant in the model is *C *[N/m^2^]. For small deformations, *C *can be derived from the Young's modulus, *E *[N/m^2^], by(2)

In the remainder of this paper, we will use Eqn. (2) and report *E *values to facilitate the comparison with *E *values in literature.

Since the intima can be relatively thick, the choice for its properties is especially important. To cover the wide intima stiffness range reported in literature, three different shear moduli for the intima were used in the current study: a high value (*E *= 1000 kPa) [25] to mimic stiff intima experimental results, a low value (*E *= 33 kPa) [28] for soft intima experimental results and an intermediate value (*E *= 500 kPa). The models generated using these three different intima stiffness values were labelled as stiff, intermediate and soft intima models. The isotropic neo-Hookean model was used for the intima since anisotropic, nonlinear material parameters for atherosclerotic human coronary intima are not available yet. The necrotic core was modelled as a very soft tissue (*E *= 1 kPa) [35].

For the media and adventitia, the anisotropic material model of Gasser et al. [36], which describes the strain energy density function for a composite material reinforced by two families of fibers, was used:(3)

where μ, k_1_, k_2 _and κ Є [0,1/3] are the model parameters. The model parameter values were obtained by fitting the material model to the experimental human coronary data [26] with the help of MATLAB (R2006b, The Mathworks Inc.). I_1 _and I_4 _= λ_θ_^2^cos^2^φ+ λ_z_^2^sin^2^φ are invariants with φ being the angle between the fibers and circumferential direction in the individual layers. All material parameter values used in the present study are listed in Table 2.

**Table 2:** Material constants of the plaque components

Tissue	Material Constants
Media	μ = 2.24 kPa, k_1 _= 65.76 kPa, k_2 _= 76.87, φ = 50.89°and κ = 0.27
Adventitia	μ = 5.86 kPa, k_1 _= 2069.42 kPa, k_2 _= 394.28, φ = 52.54°and κ = 0.20
Necrotic core	*E *= 1 kPa
Intima	*E*_stiff _= 1000 kPa, *E*_inter _= 500 kPa, *E*_soft _= 33 kPa

### Computational analysis

The finite element analyses were performed with ABAQUS (Version 6.9.1, Dassault Systemes Simulia Corp., Providence, RI, USA). The models were meshed with three-node and four-node linear, hybrid elements. Element size was chosen such that at least 5 layers of elements were present in the cap layer. The models contained approximately 100k elements. Large deformation formulation and plane strain assumption, allowing for out of plane stress build up, were used for all computational models. Appropriate boundary conditions were used to suppress rigid body motion. Static intraluminal pressure of 15 kPa (~110 mmHg) was applied as the loading condition. Postprocessing of the simulations was performed with MATLAB. The peak von Mises stresses in the cap were computed for midcap and shoulder regions (figure 2.B) separately. The 99-percentile stress [37] was used as cap stress parameter and 1% of the highest stresses was excluded. Numerical simulations with finer meshes displayed negligible changes in the stress distribution.

## Results

**Figure 3:**
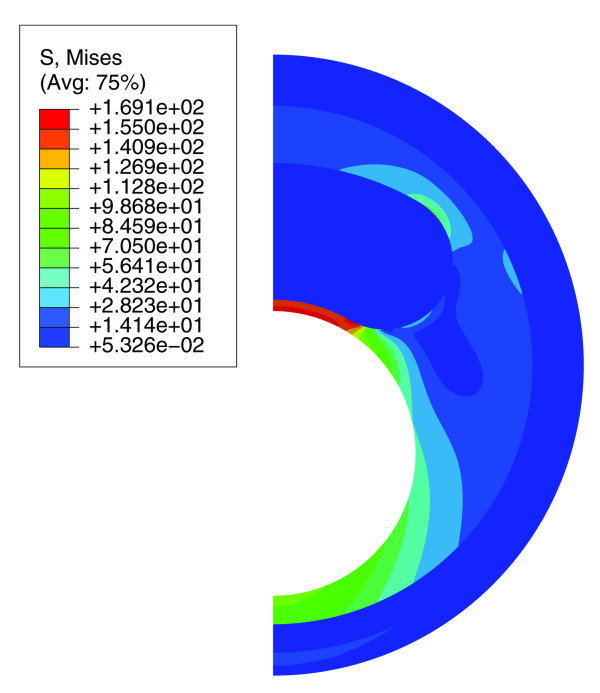
**Contour map of von Mises stresses in half cross-section of a plaque model with intermediate intima stiffness**. The highest stresses are in the cap and the peak stress values in the midcap and shoulder region are similar.

**Figure 4:**
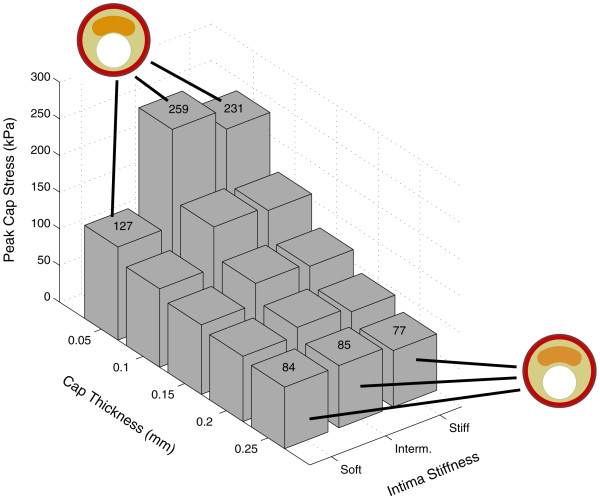
**Influence of the cap thickness and intima stiffness on the peak cap stress for the baseline geometry**. Constant parameter values for the models: necrotic core thickness = 1.2 mm, intima thickness behind the necrotic core = 0.5 mm, adventitia thickness = 0.15 mm, media thickness = 0.25 mm, necrotic core angle = 30°. Peak cap stress values and the undeformed geometries of some models are attached to the associated columns. The Young's modulus (*E*) values for the intima: 33 kPa for soft, 500 kPa for intermediate and 1000 kPa for stiff.

**Figure 5:**
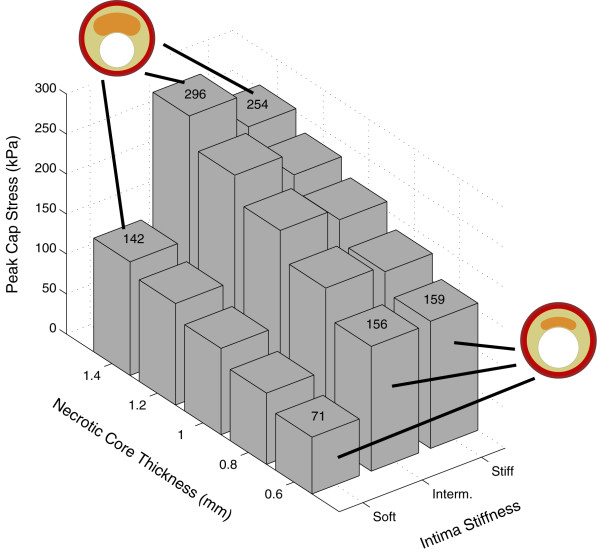
**Influence of the necrotic core thickness and intima stiffness on the peak cap stress for the baseline geometry**. Constant parameter values for the models: cap thickness = 0.05 mm, intima thickness behind the necrotic core = 0.5 mm, adventitia thickness = 0.15 mm, media thickness = 0.25 mm, necrotic core angle = 30°. Peak cap stress values and the undeformed geometries of some models are attached to the associated columns. The Young's modulus (*E*) values for the intima: 33 kPa for soft, 500 kPa for intermediate and 1000 kPa for stiff.

An example of the numerical results is shown in figure 3 where the computed stress map of a plaque half cross-section is presented. The peak cap stresses in the models mainly occurred in the midcap region. However, the difference between the peak cap stresses in the shoulder region and the midcap was small and never exceeded 5%. Generally, the results for all simulations were identical when using maximum principal stresses instead of von Mises stresses. In the remaining part of the paper, only the peak stresses in the midcaps are presented. Overall, more than 1000 simulations were performed to evaluate single and combined effects of geometric plaque parameter variations for the three intima models. The geometric variation we investigated will be illustrated by discussing two relevant examples in detail (figure 4 and 5). The main findings are presented in Table 3 and will be summarized at the end of the section.

**Table 3:** Percentage changes in the peak cap stress due to alterations in the geometric features for all intima models.

Intima stiffness	Cap thickness	Necrotic core thickness	Necrotic core angle	Intima thickness behind necrotic core	Adventitia thickness
	0.25→0.05 mm	0.6→1.4 mm	10°→40°	0.3→0.7 mm	0.1→0.2 mm
**Stiff**	+200%	+60%	- 6%	-22%	-1%
**Intermediate**	+205%	+90%	- 7%	-29%	-1%
**Soft**	+ 55%	+100%	-55%	+27%	-5%

The baseline values of the geometric features were: cap thickness = 0.05 mm, necrotic core thickness = 1.2 mm, necrotic core angle = 30°, intima thickness behind the necrotic core = 0.5 mm, media thickness = 0.25 mm and adventitia thickness = 0.15 mm. The range and direction of the variation are displayed for each feature.

The influence of the cap thickness on peak cap stress for the three different intima models is shown in figure 4. For the baseline geometry, the peak cap stress for the soft intima changed non-linearly from 84 to 127 kPa (+50%) when the cap thickness decreased from 0.25 to 0.05 mm. The intermediate intima model showed the highest peak cap stress among the three intima models for all cap thickness values and the peak cap stress increased from 85 to 259 kPa (+205%) with decreasing cap thickness. For the stiff intima, the peak cap stress was slightly lower than the intermediate intima, increasing from 77 to 231 kPa (+200%). For the models with a thin cap, peak cap stress for the soft intima was lower than the stiff and intermediate intima models, while for the models with a thick cap, similar peak cap stresses were observed for all three intima models.

Thicker necrotic core elevated the peak cap stress as well (figure 5). For the soft intima, the peak cap stress was much lower than for the other two intima models irrespective of the necrotic core size. The stiff and intermediate models showed comparable results. For the soft intima, the peak cap stress increased almost linearly from 71 to 142 kPa (+100%), for the intermediate intima, from 156 to 296 kPa (+90%) and for the stiff intima, from 159 to 254 kPa (+60%) when the necrotic core thickness was varied from 0.6 to 1.4 mm in the baseline geometry.

The maximum effects of all varied geometric parameters on peak cap stress for different intima models are summarized in Table 3. Varying the necrotic core angle of the baseline geometry from 10° to 40°altered the peak cap stress by -6 for the stiff intima model and by -7% for the intermediate intima model. However, the change was -55% for the soft intima model. Due to change in the thickness of the intima layer behind the necrotic core from 0.3 to 0.7 mm, the peak cap stress decreased by 22% for the stiff intima model and 29% for the intermediate stiff model. By contrast, the peak cap stress was elevated by 27% for the soft intima model. For the stiff and intermediate intima models, change in the adventitia thickness from 0.1 to 0.2 mm had almost no effect on the peak cap stress (-1%) and for the soft intima model, the peak cap stress changed only by -5%. Local regression of the media layer did not change the peak cap stress more than 1% in any of the intima models studied (data not shown).

## Discussion

Insights into biomechanical factors that influence cap stresses of vulnerable plaques are important for rupture risk prediction. The morphology of an atherosclerotic plaque and the material properties of its components are important determinants of the stress distribution in the plaque cap. The present study showed that the stiffness of the intima of a plaque has a profound influence on the resulting cap stresses. It also revealed that, in addition to the thickness of the plaque cap and necrotic core, other morphological plaque features as necrotic core angle and intima thickness behind the necrotic core are of great importance for cap stresses.

The experimental studies reported stiffness values ranging from 30 kPa up to 2000 kPa for atherosclerotic intima [19,21-26]. All previous computational studies used stiffness values in the upper range of the reported values for intima. In some studies [12,13,18] the impact of the material stiffness of intima on peak plaque stress has been investigated. However, even the lowest stiffness values in these papers were much higher than 30 kPa. Recent studies [28,38] that focused on measuring the material properties of intima tissue have confirmed a previous study from Lee et al. [27] indicating that low stiffness values for the intima might be more appropriate. To the authors' knowledge, this is the first study that used intima stiffness values in the lower range of the reported experimental values [27,28] and investigated how cap stresses are affected by intima stiffness.

For the stiff and intermediate intima models, the main load bearing plaque structure was the intima. Therefore, the stresses in the intima were higher than the stresses in the media and adventitia layers. For the soft intima models, the media and adventitia were relatively stiffer than the intima and contributed to supporting the overall load. Consequently, stresses in the media and adventitia increased while the ones in the intima and cap decreased. Hence, the soft intima models usually showed much lower peak cap stresses than the stiff and intermediate intima models.

Intima stiffness also altered the effects of geometric variations of the plaque morphology on peak cap stress. For the stiff and intermediate intima models, cap thickness was the most essential geometric plaque parameter within the investigated parameter range. A thinner cap elevated the peak cap stress dramatically, which is in line with the previous studies [8,18,39]. Necrotic core thickness was also an important geometrical feature, and an increase in the necrotic core thickness resulted in higher stresses, confirming the findings of Ohayon et al [16]. Since the intima was the main load bearing structure, a thicker intima behind the necrotic core contained the deformation of the cap, thus the peak cap stress was reduced.

Since the media and adventitia were the load bearing structures for the soft intima models, the effect of reducing cap thickness was much less pronounced. The necrotic core thickness appeared to be the most influential morphological plaque parameter. A thicker intima layer increased the peak cap stress because deformations of the necrotic core and cap were much larger due to the larger soft intima behind the necrotic core. Furthermore, necrotic core angle emerged as an important geometric parameter in the soft intima models, being equally important as the cap thickness. This finding can be attributed to the difference of the circumferential length of the cap in the midcap region. We observed that the displacement of the central part of midcap region in radial direction was comparable for all the necrotic core angles. The length of the cap for smaller necrotic core angles was smaller than the length of the cap for the larger necrotic core angles. When subjected to comparable radial displacements, the smaller cap length for the smaller necrotic core angles induced higher strains and therefore higher stresses.

Plaque rupture is an interplay between the stress in the cap and local cap strength. To assess the risk of plaque rupture in clinical setting, both need to be determined. We showed that the stresses in the cap of a vulnerable plaque strongly depend on material properties of the intima and the geometric features of the individual plaque components. Currently, intima-media thickness (IMT) is used as a clinical measure to both detect and track the progression of atherosclerosis. IMT corresponds to the sum of the thickness values of the cap, necrotic core, intima behind the necrotic core, and media in the present study. IMT has been shown to correlate with the presence of coronary disease, and predict cardiovascular events [40], and a larger IMT was observed more in unstable angina patients than in the stable angina patients [41]. However, we could not find any relation between IMT and peak cap stress in the current study (data not shown). The results of the current study support the need for more detailed quantitative imaging of the individual plaque components for stress assessment in coronary plaques. A combination of an intravascular ultrasound derived imaging technique [42] and optical coherence tomography (OCT) [43] could potentially provide the necessary quantitative geometric data for various plaque components, including cap thickness and necrotic core size. Rupture risk assessment also requires local information about cap strength. Several factors influence tissue strength, including local macrophage density [44]. The presence of macrophages can be detected in vivo by means of OCT [45]. Although further clinical validation of these advanced invasive imaging techniques is required, they might be combined in clinical imaging tools for rupture risk assessment.

One of the main limitations of the present study is the relatively simple material model that was used for the intima section. Anisotropic, nonlinear material properties found in literature were used for media and adventitia. However, intima tissue was assumed to be isotropic since no data is available for anisotropic material parameters for atherosclerotic human coronary intima. Atherosclerotic lesions are often highly complex structures with irregular geometries (figure 1). Due to the smooth tissue interfaces and component morphologies in the idealized geometries, no local stress concentrations were observed in this study. This explains the similar stress results in the shoulder and midcap region in contrast to some previous studies that used real plaque geometries [12,13,15]. However, the idealized geometries allowed isolating the effects of the investigated geometric features. In the current study, 2D models were used and stress results might be different than those of 3D models. However, Tang et al. [46] reported similar stress distribution maps for 2D and 3D finite elements models of atherosclerotic plaques. 2D models are easier to construct, require less computational effort, and are therefore effective for parametric analysis. Initial stresses were not incorporated in the computations. For the computations with realistic geometries, initial stresses should be taken into account since this leads to physiologically more realistic strains and stresses. It is likely that including initial stresses would not change the main findings of this study since idealized geometries were used and relative changes were evaluated. Residual stresses were not incorporated in the models either. Ohayon et al. showed that residual stresses in atherosclerotic plaques change stress values, but not the distribution within the structure [47]. Thus, it is likely that incorporating residual stresses would not change the main findings of this study. Another limitation is that calcifications within atherosclerotic plaques were disregarded although they are present in atherosclerotic lesions [1]. Although large calcifications might lower the plaque stresses [14], several studies have shown that microcalcification might induce local stress concentrations and elevations [48,49]. If and how the impact of calcification on cap stresses is altered by intima stiffness and geometric features warrant further studies.

## Conclusions

For the stiff and intermediate intima models, cap thickness was the most important morphological risk factor. For the soft intima models, necrotic core thickness and necrotic core angle had a bigger impact on peak cap stress. We therefore need to enhance our knowledge of intima material properties if we want to derive critical morphological plaque features for risk evaluation.

## Competing interests

The authors declare that they have no competing interests.

## Authors' contributions

Study design: ACA, LS, HB, MAG, RV, AL, AFWS, JJW, FHG

Computations: ACA, LS, FHG

Data analysis and interpretation: ACA, LS, JJW, FHG

Manuscript preparation: ACA, LS, JJW, FHG

Manuscript editing: ACA, LS, HB, MAG, RV, AL, AFWS, JJW, FHG

Manuscript revision: ACA, LS, HB, MAG, RV, AL, AFWS, JJW, FHG

All authors read and approved the final version of the manuscript.
